# Beyond Scientific Mechanisms: Subjective Perceptions with Viniyoga Meditation

**DOI:** 10.3390/ijerph16122200

**Published:** 2019-06-21

**Authors:** Carrie Heeter, Marcel Allbritton, Chase Bossart

**Affiliations:** 1 Department of Media and Information, Michigan State University, East Lansing, MI 48824, USA; 2 Core Resonance Works, New Orleans, LA 70119, USA; marcel.allbritton@gmail.com; 3 Yoga Well Institute, Mill Valley, CA 94942, USA; chase@yogawell.com

**Keywords:** meditation, yoga, ethnomedicine, Viniyoga

## Abstract

Healthcare professionals and research scientists generally recognize the potential value of mind–body practices grounded in ancient wisdom, but often have limited direct experience with such practices. Meditation participant self-reports provide a window into subjective experiences of three Viniyoga meditations and how and why those meditations could contribute to health and well-being outcomes. Each of the meditations in this analysis had a unique structure and used a different aspect of the ocean as a meditation object. Yoga philosophy and yoga anatomy models of the human system are used to help explain participants’ experiences and associated personal benefits and insights. Four aspects of the individual that can influence what happens for them in meditation are illustrated with tangible examples: (1) What is happening in generally in someone’s life; (2) the state of their system (mind, body, breath) around the time of the meditation; (3) reactions to the meditation steps and instructions; and (4) their prior experiences with the object of meditation. Summaries of the practices, and why and for whom each meditation might be beneficial are discussed. The authors’ perspectives are grounded in Viniyoga and yoga therapy.

## 1. Introduction

Yoga and meditation are popular forms of self-care. In 2017, 14.3% of U.S. adults reported practicing yoga and 14.2% reported using meditation [1]. The rising popularity of yoga and meditation has been accompanied by a proliferation of styles and approaches. Approaches to meditation include mantra meditation, mindfulness meditation, transcendental meditation, guided imagery, progressive relaxation, and spiritual meditation as well as meditation that is part of yoga, tai chi, and qi-gong [2]. Millions of people have tried one or more forms yoga or meditation taught by different teachers. There are at least 22 different styles and schools of yoga in the west [3]. As a result, there are endlessly divergent understandings of what meditation is and how it works.

Viniyoga is an approach to yoga for health and well-being that begins with the premise that yoga is “intimately personal and deeply experiential” [4] (p. 10). Viniyoga prioritizes the importance of adapting the yoga to the person [4,5]. Yoga in the west often emphasizes physical postures. However, meditation is one of the tools of yoga (along with others including breath, movement, mantra).

The wisdom of yoga has historically been passed down from teacher to student. Viniyoga is grounded in the ancient Yogasutras of Patanjali [6] through the lineage of Śri T. Krishnamacharya [7] as passed on to his son Mr. TKV Desikachar [8]. All three authors of this article are trained in this tradition. Bossart was a personal student of Mr. Desikachar (now deceased) for more than two decades, including four years of study with him in India. Allbritton has been a personal student of Bossart for 15 years. Heeter has been a personal student of Allbritton for 7 years.

Why is Viniyoga meditation considered a form of ethnomedicine? Why would attention-based practices impact health and well-being? The five dimensional Pañcamaya model in Indian anatomy (from the Taittrīya Upanisad) explains that the body, breath, mind, personality, and intellect are overlapping dimensions of the human system [9,10]. Changes in one dimension affect the other dimensions. For example, when the mind is agitated, bringing attention to the breath and gradually slowing the breath will tend to calm the mind. Connecting with the feeling of being in a favorite place in nature through meditation can elicit many of the same physical and emotional reactions of actually being in that place, with associated health benefits. For example, meditating on a large tree every day over a period of several weeks might help someone who is anxious feel more grounded. Meditation can strengthen desirable patterns and it can produce insights about oneself and life situations.

An object of meditation is where the attention is placed. Meditation objects are a little like portkeys in the fantasy world of Harry Potter, that when touched by a person immediately transport them to a specific place [11]. Like touching a portkey, focusing attention on a meditation object can transport the person to a meditation experience. An object of meditation can be almost anything: tangible objects in the natural world (such as the moon or a lake), subtle qualities or feelings (such as gratitude or safety), ideas, aspects of the body (such as the breath), or of the self (such as one’s tendencies or activities of the mind) [6,8,12]. The object of meditation is often chosen to help a particular person develop a desirable new pattern or strengthen an existing pattern. Developing patterns through meditation typically involves systematic repetition of meditation experiences with the same object over a period of time.

Each individual has a unique body and mind, a unique personality, ways of thinking and reacting, memories, and life circumstances. Also unique is how an individual responds to a meditation and what meditation objects are useful and appropriate for them. Yoga therapy is a form of ethnomedicine in which highly trained yoga therapists assess a yoga therapy client’s needs and design a personal daily practice for the client to do, selecting and sequencing appropriate tools of yoga (such as movement, breathing, meditation, mantra) [4,13,14]. A yoga therapist observes the client over time and adapts the practice as the client changes. Scientific research on yoga as therapy is reviewed in *The Principles and Practice of Yoga in Health Care* [15]. The volume includes state of the art literature reviews, with 17 chapters each devoted to a specific health condition.

Choosing an appropriate meditation object for an individual requires training, experience, and observation. Mr. Desikachar compared meditation to rain [12]. If rain falls on the ocean, not much happens. Rain falling on rock or dry hardened soil runs off. Rain on saturated soil may destroy crops. On freshly tilled soil, rain may provide needed moisture for seeds to grow. A meditation is like the rain. The individual is like the soil. Viniyoga teachers and yoga therapists seek to understand the individual (the soil) and design a practice (the rain) that supports the individual. What happens in a meditation depends on the individual and their circumstances.

Whether experienced as a personal daily practice or part of a group yoga class, meditation in the tradition of yoga typically involves a sequence of steps that incorporate different tools of yoga. The early steps often involve movement patterns and breathing techniques that, along with specific functions of the pattern or technique, generally serve to prepare the mind for meditation. Meditation can be introduced at the end of the practice. Or the meditation object can be called to mind at the outset, followed by movement and breathing, perhaps revisiting the meditation once or more as the state of the human system changes throughout the practice. The possibilities for how a practice is designed are endless. Choices are guided by understanding of the techniques and how to combine them, the goals of the meditation, and understanding of the needs, capabilities, and limitations of the individual(s) who will be doing the practice. Thus, meditation is a designed experience, including selection of meditation object and selection and sequencing of steps.

This article addresses gaps of understanding that impede scientific research on yoga and meditation. A search of the PubMed online database conducted by the authors in May 2019 found that more than 500 scientific articles about yoga and/or meditation interventions for health, and more than 700 reviews and meta-analyses have been published in the last 5 years. Published research on meditation interventions for health often fails to include adequate description of the meditation intervention being studied [16]. Meta-analyses of the effects of yoga and meditation can further impede scientific understanding by grouping studies of disparate meditation interventions together in their analysis [16]. Readers of scientific research about meditation interventions cannot begin to understand the intervention being studied if the intervention is not clearly described. Even if the intervention is described in detail, a verbal description of a meditation intervention does not convey the experience of practicing that meditation.

The amount of time and commitment that is required to deeply study and practice a single meditation tradition, combined with assortment of traditions, presents steep barriers for research on meditation as ethnomedicine. Having direct experiences with meditation contributes to understanding the phenomenon. Personal experience with meditation can range from cursory exposure of having tried meditation a few times to intensive exposure through attending retreats and maintaining a daily practice over many years. Personal experience with a specific meditation approach can be extensive, and may include working with a mentor, reading books and articles, studying the yogasutras, and completing yoga teacher training or yoga therapy training.

Public health and healthcare professionals, research scientists, and the general public who have professional or personal interest in meditation for health and well-being can benefit from reading first person perspectives describing what it is like to experience Viniyoga meditations. The accounts reported in this article showcase personal experiences of meditation. Self-reports of subjective experiences with meditation are used to illustrate how and why each individual who is meditating engenders a personalized meditation experience and outcomes. Quotes from long-time students and teachers in the Viniyoga tradition describing their experiences show how identical meditation guidance resulted in unique personal experiences. 

Participants’ experiences with three different meditations are described and analyzed as examples of how meditation in the tradition of Viniyoga and yoga therapy functions to support health and well-being. Each practice has a different structure, each uses different aspects or qualities of the ocean as a meditation object. Details of the practices, details of the objects of meditation, and why and for whom the meditation might be beneficial are discussed. Excerpts from instructor-led discussions with advanced yoga students who did the meditations are used to explain how the individual interacts with meditation instructions to form a personal experience of the meditation. The analysis focuses on four aspects of the individual that influence what happens for them in the meditation: (1) What is happening generally in someone’s life; (2) the state of their system (mind, body, breath) around the time of the meditation; (3) reactions to the instructions or steps in the meditation; and (4) their prior experiences with the object of meditation.

## 2. Materials and Methods

Yoga Well Institute Director Chase Bossart teaches a weekly half-hour online meditation class, followed by a bi-weekly Therapeutic Applications of Meditation online class. Students describe their experience with the meditation and Bossart relates those experiences to principles of yoga and the design of therapeutic meditation. Students in these classes are long time practitioners and students of Viniyoga. Each has a mentor and personal daily yoga practice (which often includes meditation). Most have been participating in Bossart’s bi-weekly online Therapeutic Applications of Meditation class for years. All are either training to become or are already working as yoga therapists or yoga or meditation teachers.

Each week, students experience a new meditation, with unique variations of meditation object, structure, and yoga techniques. By observing themselves during the practice, students discover what works well for them and what works less well. They gain self-knowledge and an experiential understanding of how to design and teach their own classes. The meditations are meant to demonstrate many different approaches to creating a meditation practice, and to show how these practices might be used to support health and well-being.

For two consecutive months in 2016, Bossart used some aspect of the ocean as the meditation object for his weekly online meditations. The source for the self-reports was a set of four of Bossart’s 90-minute instructor-led discussions about eight meditation experiences—the one participants had just completed and the meditation from the previous week. Those discussions were wide-ranging, including calling on participants to describe their experiences, conversations about sequencing and specific movement or breathing steps, and about yoga philosophy.

The transcripts were merged and entered into NVivo, a qualitative data analysis tool. The complete 27,968-word document was coded by participant, by meditation, and by various constructs related to Viniyoga and yoga philosophy. After completing the coding, the authors changed course when they realized the self-reports could be a vivid means to show people with expertise or interest in meditation, but no personal experience with Viniyoga meditation practices, what these experiences are like.

Grouping participant comments based on factors that influenced how and why they experienced the meditation was considered. Although this approach supported the importance of those factors, the presentation lacked the context of the meditation practice participants were referring to. With that goal of illustrating unique personal experiences with Viniyoga meditation, the authors recognized a need to introduce the meditation practice and to present participant experiences in the context of the practice. There was a concomitant need to reduce the number of different meditations for the sake of clarity and focus.

The majority of each class discussion focused on the meditation participants had just completed. So that it would be easier for readers to keep the meditation being discussed in mind, comments about “last week’s meditation” were eliminated. Early versions of this article introduced all four current week practices followed by synthesis of participant comments. However, describing four different practices in detail seemed to obscure the impact of the examples. Limiting reporting of findings to three of the four practices allowed the manuscript to touch on the main points for the intended analysis while reducing redundancy and forestalling potential reader fatigue.

Table 1 shows pseudonyms of nine participants and which meditations they commented on. Anonymized transcripts from the online class discussions and diagrams and detailed descriptions of the steps in the meditations that were discussed are available from the corresponding author’s website. See Supplementary Materials for access details.

The Michigan State University (MSU) Office of Regulatory Affairs Human Research Protection Program determined the study to be exempt under the MSU Flexibility Initiative Exemption Category 97. Discussion participants provided informed consent for their statements made during the ocean meditation discussions to be included in the research.

## 3. Results

For each ocean meditation and discussion, results begin with an overview table summarizing the experience of the meditation including why and for whom that meditation might be beneficial. The participant experience subsections compare and contrast unique individual experiences of that meditation. The order of participant comments was rearranged to facilitate narrative flow of the main points the article seeks to convey. The authors added explanations and clarifications to compare, contrast, and link participants’ direct quotes.

To further help readers associate participant comments with the factors highlighted in this article that can influence their meditation experience, codes are embedded within the dialog or explanation. L^IFE^ is the code for what is going in generally in someone’s life. S^OS^ is the code for the state of their system (mind, body, breath) around the time of the meditation. I^1,2,3^ flags reactions to the meditation steps and instructions. And O^BJECT^ refers to their prior experiences with the object of meditation. These four influences can enhance or distract from the meditation experience, indicated by a “+” or “–“ in front of the code.

### 3.1. Calm-Ocean-Depths Meditation

#### 3.1.1. Meditation Overview

Table 2 summarizes the steps in three main sections of the calm-ocean-depths meditation, organized by preparation, main meditation, and the closing step which re-visits the main idea of the meditation, but in a different way.

#### 3.1.2. Why and for Whom Might this Meditation be Beneficial?

This meditation began by seeding the feeling of “I am calm” at the end of each breath throughout the preparation steps. Then the sense of the deep ocean, very still, was introduced, implicitly mapping calm onto the metaphor of the deep ocean. As discussed in the introduction, meditation objects function like a portkey, to generate an experience. Using the deep still ocean as a metaphor for the feeling of deep calm can help generate an experience of deep calm that is more tangible and accessible than simply trying to feel deeply calm.

The meditations in this article were designed to teach about meditation and participants experienced each meditation only once. If a meditation similar to the deep calm ocean meditation were used for health and well-being, the person would do the same meditation daily over a period of time. Repeated experiences with associating deep ocean with deep calm and time spent feeling deep calm in the meditation would strengthen the pattern of calm. Over time as this pattern becomes more established, the person might, when encountering stressful life situations, briefly call to mind the deep ocean, which now serves as a portkey to feelings of calm.

Next the instruction was to allow attention to rise to the surface where a giant storm is coming, and then to cycle through two or three rounds of still deep ocean to stormy surface to have a sense of the contrast. Doing this part of the meditation gives people an experience of shifting their attention from storm to deep calm.

For some participants, doing this meditation may transfer to daily life. They may make a connection between what they do when they encounter challenges in daily life (storms) and staying in touch with their own deep inner calm.

One of the goals of the instructor in using this particular meditation for teaching about meditation was to give students an experience with complex instructions. Complexity can be very helpful for some people for keeping the mind engaged. Complexity can be unnecessary or frustrating to others. In the paragraph below we describe the instructions in detail.

Step 2 of the meditation introduced breathing technique that involved slightly tightening the throat on exhalation. Step 3 added the practice of moving the tip of the thumb up the index finger on inhale and moving the tip of the index finger down the thumb on exhalation. Step 4 added an instruction to move the thumb from finger to finger after each breath (while continuing to move the tip of the thumb up the finger on inhalation and down the finger on exhalation) and added silently repeating “I am calm” or “calm calm” during a pause after exhalation. Step 5 continued the breathing pattern, the thumb movement, and the silent mantra during pause after exhalation and added raising one arm up from the front on inhalation and lowering the arm on exhalation, alternating arms. Step 6 asked participants to notice “where did the mind go?” Step 7 resumed the sequence but now on inhalation one arm is raised up from the front, on exhalation bend forward over the knees, on inhalation, return to an upright position raising the other arm up.

Following all of these instructions takes a lot of attention. Preparation involving this level of complexity would not be given to people who are not experienced with yoga. Meditation instructions that ask someone to do something beyond what they are capable of doing will not be effective.

#### 3.1.3. Participant Experiences

In the authors’ tradition of meditation, enough instruction is given to set a general direction for the meditation, but room is purposefully left for participants’ own experiences to unfold. In this example meditation, the teacher does not offer details of what the deep ocean or the surface storm look like. Each person’s memories and imagination fill in the details. Life events and how they are doing at the time of the meditation colors (influences) what happens for them in the meditation.

Four of the six women who shared their experiences with the calm-ocean-depths meditation spontaneously connected the storm with events going on in their lives, even though there were no explicit instructions making that connection. For Alice, at first the visualization was just being deep in the ocean and then coming to the surface and seeing the storm. “But quickly it became very personal for me about what’s happening in my life (+L^IFE^). It was easy for me to actually feel the energy of where the storm is and where the calm is in my body”.

Even though Iris had never been deep in an ocean, imagery came to mind. “I’ve never been that deep in the ocean, but a deep, deep blue came into my mind. On the surface the storm was approaching. The clouds were super dark and the waves were pretty stormy”. Like Alice, Iris immediately associated the calm and the storm with things going on in her life. “[The meditation] was very reflective of my life experiences [of surface storm and deep inner calm] (+L^IFE^)”. Even when her attention was on the surface with the storm, Iris felt detached from the storm. “For some reason I wasn’t part of that storm. I was in it, but I wasn’t of it. Every time I repeated the exhale with ‘I am calm,’ it brought me back [to calm]”.

What showed up for each participant was personal and unique throughout the meditation, even though each was following the same meditation instructions. The overlapping dimensions of each individual (body, breath, mind, personality, and intellect), their memories, and current activities interact with the meditation instructions given by the teacher to engender a unique personal experience of the meditation. Iris imagined being surrounded by deep blue in the deep ocean depth and very dark clouds on the surface. Alice’s deep ocean and surface storm would have looked and felt very different than what Iris experienced. What was going on in Alice’s life that felt calm or storm-like would have been completely different than what was going on in Iris’ life that felt calm or storm-like.

Similar to Iris reporting she was in the storm but detached from it, Amruta felt unaffected by the turbulence. There was not an actual storm in Amruta’s meditation experience, there was only turbulence. The meditation instructions specifically said “allow attention to rise to the surface where a giant storm is coming”. And yet, for Amruta there was no storm. Amruta “could easily glide down to the deepest part of the ocean. The water was clear. I was sitting there. Then I came up, there was no storm. There was turbulence. There were waves and I was floating. I connected the turbulence to my past experiences—the times when I was able to be calm and the times when I was in the turbulence. But I saw myself floating because now I see myself [in my life] not being adversely affected by turbulence (+L^IFE^)”.

When Amruta began the meditation, she was directing her attention to follow the meditation instructions. Then a shift happened, and she could feel herself gliding down to the deepest part of the ocean and sitting there. These details came from her. They were not in the instructions. When the instructions said to rise to the surface where a giant storm was coming, there was no storm. Amruta’s mind seamlessly accepted the appearance of turbulence rather than a storm. She was along for the ride, watching what showed up as a result of focusing on the meditation object.

Lena’s experience of the storm changed each time she came up to the surface, with the storm feeling less intense and less threatening with each encounter. The first time Lena came up to the storm on the ocean surface, “it was like a movie with the giant wave that comes in and flips the boat over and I actually experienced fear. I could easily go back down. Each time I came up I could still see the storm but it was less and less of a visceral feeling”. There were no instructions in the meditation suggesting that the storm would become progressively less threatening. For Lena, that was the experience that showed up. Metaphorically, the practice of reacting to life’s inevitable “storms” less viscerally can be a useful shift in perspective.

Toward the end of the meditation, Lena had insight into times in her work where she was operating from a sense of deep calm, and times in her work when it felt like she was in a storm (+L^IFE^). In Step 10, Lena linked the depths of the ocean to her day yesterday when she “felt fully present with clients and holding space for them successfully. And then to be in the storm my notes and all of my lists were spread all around me and my mind was racing and grasping for answers [from my trainings]”. She was able to link the feeling in her body during the deep calm work experience and the stormy work experience. What happened for Lena in the meditation may be useful to her in daily life, as she learns to recognize when she is working from deep calm and when she is caught in a storm. And she may be able to notice when she is caught in a storm and stop, perhaps making different choices.

Neither Jade nor Kat connected the storm with challenges in daily life. Neither had a realization during the meditation that “even when my life is stormy, there is a deep part of me that is calm”.

When Jade heard the instruction to “have the sense of deep ocean—very still”, the idea brought up a lot of fear (–I^1,2,3^, –O^BJECT^). Rather than having an experience she knew would be unpleasant, Jade looked for an alternative to being in the deep ocean. “I was thinking where can I go [instead]? Where can I go that is ocean-like but safe? I had been in Barcelona and there was a park by Gaudi with a pavilion that has columns to look like you’re under the ocean. So I went there and hung out. For the storm part I imagined pouring rain and storming outside of the pavilion. I could stay inside this nice safe harbor in this wonderful structure”.

Was Jade’s meditation a success or a failure? She did what Bossart refers to as “operating your vehicle responsibly”. If an instruction, whether breathing, movement, or meditation, does not feel right, do not do it. Modify the instruction to be right for you. Jade asked her mind to come up with a place that was like the deep ocean but safer. A creative solution came to her—a playful ocean-like park pavilion by Gaudi.

Jade’s experience appears to have had a stronger emphasis on safety (the safe harbor) than on deep calm. The structure also protected her from the storm. She felt protected from the storm, but she did not have the experience of deep inner calm even when there is a storm on the surface. She did not experience a juxtaposition of deep inner calm and surface storm, but she experienced different benefits. The experience of modifying the instruction to stay safe is practicing agency and proper operation of “her vehicle”. Connecting with the feeling of safety and being protected through meditation can strengthen these patterns and contribute to reduced stress and improved well-being.

To Kat, meditating on being calm felt like broccoli [it’s good for you, you’re supposed to want to be calm] (–O^BJECT^), whereas the wild storm was glorious and fun [like chocolate] (+O^BJECT^). She “loved the storm. The power of it. It felt so tempting and interesting”. Kat did not make the same connection others did, to the storm representing difficulties in life. She “became the storm” and enjoyed the feeling, rather than imagining her human self in a storm. For Kat the storm was not a dangerous force or threat to her safety. Kat WAS the storm.

Was Kat’s meditation a success or a failure? During the discussion after the meditation, Kat remembered that she had completed her daily yoga practice and was already very calm right before the ocean meditation began. For the first 20 minutes of the meditation the instructions focused on techniques to help bring breath, body and mind toward calm. The resistance Kat felt toward the deep calm she believed she was supposed to prefer was probably accurate awareness that the meditation was taking her too far into calm, given the very calm state of her system already (–S^OS^). When the mind is heavy or dull the mind is less directable.

Being the storm moved Kat out of feeling torpid. Loving being the storm also reinforced a pattern of intensity and overload which is not necessarily something she needs more of. She may have gained some potential insights—(1) there is such a thing as too much calm; and (2) storms tempt me (but may not necessarily be good for me). If Kat had done this meditation at a different time rather than shortly after her personal meditation practice, she might have welcomed and benefitted differently from the deep calm, and she might not have felt pressured to value calm more than storm.

The imagery and associations that arose during the calm-ocean-depths meditation were distinct for each participant (Table 3). Everyone’s deep ocean was distinct. Everyone’s storm was distinct. For some the storm was literal. For others, during their meditation experience, the storm became a metaphor for life’s challenges. The qualities of the storm and the participants’ relationship to the storm often changed during the meditation.

### 3.2. Ocean Tides Meditation

#### 3.2.1. Meditation Overview

Table 4 summarizes key sections in the ocean tides meditation structure, starting with preparation, followed by the main meditation, and a closing step to complete and prepare to transition out of the meditation.

#### 3.2.2. Why and for Whom Might this Meditation Be Beneficial?

The metaphor of incoming tide bringing nourishment and outgoing tide cleansing is mapped onto inhalation and exhalation throughout the half-hour meditation. Cleansing of the body is necessary to sustain life. Cleansing happens physically with the exhale (releasing CO_2_). Metaphorically, cleansing of the body and mind is associated with elimination of waste, washing away dirt or unpleasant events or memories we are holding on to. Linking each exhalation with the idea of cleansing can help rid the body and mind of unwanted physical tension or emotional residue.

If readers of this article find themselves feeling skeptical that a meditation associating the outgoing ocean tide with cleansing could release tension in the body and mind, remember that the idea that the body and mind are interconnected is a central premise of mind–body practices. What someone does with their mind affects the body. In meditation, metaphor is the language of the human system.

A meditation where each exhalation is associated with a cleansing ocean tide going out is likely to be more effective than telling someone to just stop worrying about something that is really bothering them. Even if an individual somehow manages to simply notice negative ruminative thoughts and let them go, the other ways their human system is holding on to worry (stress hormones, clenching muscles, elevated blood pressure, etc.) would still be happening. Neither the mind nor western medicine knows all of the ways a particular individual’s body is holding on to a painful or stressful life event. Meditation like the ocean tides allows the human system to feel cleansed, without specifying particular aspects of the body or mind to cleanse. For a cleansing meditation to have a sustained impact, the meditation would need to be repeated daily over a period of time.

Like cleansing, nourishment of the body is necessary to sustain life. Inhaling is physically nourishing—it brings oxygen into the body. Emotionally we may feel nourished by a vacation, or evening with friends, or a good book. Metaphorically, the mind can also be nourished by a diet of nourishing experiences. In the meditation, linking each inhalation with feeling nourished practices allowing the feeling of being nourished to come in.

Many people have been trained by their early developmental experiences that being nourished is dangerous. Perhaps it was not safe to accept nourishment. Or perhaps there is a feeling of not being worthy of nourishment. When you give someone with issues around nourishment a meditation that involves being nourished, the visualization can get sabotaged in some way. For some people the water might be dirty. People may encounter dangerous objects in the water. The water may actually have blood in it in the extreme. When that happens it is a sign that the metaphor needs to be changed in the meditation. In yoga for healing, the meditation is customized and adapted for the individual. One size does not fit all.

#### 3.2.3. Participant Experiences

Amruta’s brief description of her experience touches on all four of the influences on a meditation experience that this article focuses on: (1) what is happening generally in her life; (2) the state of her system (mind, body, breath) around the time of the meditation; (3) reactions to the meditation instructions; and (4) her prior experiences with the object of meditation. As far as prior experience with the meditation object, Amruta mentioned that she loves the ocean, it’s the place she goes to be by herself (+O^BJECT^). In Amruta’s meditation experience, “the water is all clear. The nourishing and the cleansing both worked for me”.

However, things going on in her life (+L^IFE^), and lack of sleep the night before affected Amruta’s meditation experience (–S^OS^). “I’m very anxious about some things that I’m going toward right now and my mind is crowded. I slept disturbed and wasn’t sure if I wanted to wake up to do this class or not. I’m glad I did because it just worked. [Feeling] the tide going in and out was more about my breath [than about visualizing the tide]. Stillness wasn’t possible today without dropping into my breath because my mind was crowded”.

Because she was feeling anxious and tired, Amruta was not able to visualize the tide going in and out and to link the tide with nourishing and cleansing (–I^1,2,3^). As an experienced meditator, she was able to connect with the underlying point of the meditation (to feel nourished and cleansed). Rather than being upset about failing to visualize the tide, she relaxed into the breath and the feelings.

Lena’s mind was involved in directing the meditation. Some of the time, she could be with the meditation. She observed a rhythm that developed with the breath and the tide coming in and out. “I could really appreciate the nourishment. And I could really appreciate the cleansing”. At other times, the meditation instructions did not fully make sense, causing her logical mind to get caught up mentally thinking about “well, tide doesn’t come in and out every wave”. At that point the mind was thinking about the instructions, not having the experience (–I^1,2,3^). She came up with a way to get back into the meditation. “But I surrendered to [the instructions]. Oh, the tide is full. The tide is way out. I have really neat memories of watching the tide really far out (+O^BJECT^). There’s a sense of space”.

At step 6, Lena again had trouble trying to comply with the instruction and she moved out of just doing the meditation into frustration and self-evaluation. Her mind was activated (–I^1,2,3^). “When the instructions added hold after inhale and exhale, I got frustrated thinking I don’t have the capacity breath-wise to maintain the tide being in and the tide being out. So at that point I was into my head and out of the experience”. Also in step 6, the option was given of having several breaths with the tide coming in and several breaths with the tide receding. This choice brought Lena out of experiencing and into thinking. “At first I stuck with changing every breath but then I tried the option. I think giving the option of changing [the timing of breath and tides] in the middle was distracting for me”.

Overall, doing the meditation gave Lena experiences of feeling nourished and feeling cleansed some of the time. However, the instructions in this meditation such as having the tide go in and out with each breath and then holding at the end of each breath caused Lena’s attention to move to thinking about the instructions rather than to following the instructions. As an experienced meditator, Lena was able to return to the meditation experience after the disruptions. However, different instructions would have been better for her.

As was discussed in the introduction to this ocean tides meditation, the idea of accepting nourishment can be challenging to some people. Resistance to accepting nourishment can show up in the details of how a meditation unfolds for the individual. Penelope noticed fish in the water. “At the very beginning of the tides and the waves, little fish came and visited me. But that was fine. They just came in. I thought, oh wow, check it out. Little fish. And then they went out with the next wave”. She noticed the fish, but they did not interfere with her meditation experience.

Like Lena, Alice felt confused about how to map tides going in and out with the breath. Was the tide for each breath a little bit higher across several breaths, or did the entire tide change happen in one breath (–I^1,2,3^)? Then she heard the instructions of incoming tide being associated with nourishing and outgoing tide with cleansing. For a while it felt great but then she started thinking, “all these little fishes. What am I going to do with these fishes?” For Alice, meditations with visualizations sometimes go too far. Little details show up and then the primary intention of the meditation is missed.

As the meditation experience continued, Alice found herself “trying to figure out what was nourishing in the wave”. So instead of experiencing the feeling of being nourished, her mind was busy thinking about why waves might be nourishing. When the mind gets involved in thinking about the instructions, it often can derail a meditation, pulling the meditator’s attention away from the meditation object.

Jade is working to develop her capacity to feel nourished in her personal practice, noting that it’s a challenge (–L^IFE^). This showed up in her meditation experience. For Jade the water “wasn’t completely clean. I didn’t see ocean water as very ideal to bring in. So instead I focused more on the cleansing [part of the meditation]. The tide came, sweeping all the garbage back out leaving a nice clean beach”. As an experienced meditator, Jade noticed that bringing in somewhat dirty ocean water was not going to be very nourishing, so she shifted her focus in the meditation to concentrate on cleansing.

The way a meditation is designed can interfere with the intended experience for some people, while being very helpful for others. One size does not fit all. Like Jade, Kat is also working on feeling nourished in her personal practice (+O^BJECT^). In the ocean tides meditation, “having the inhale be nourishing, which is a new and tentative feeling for me, and then the exhale switch to cleansing was very useful because it would give me a break. I would pull a little nourishment into myself [on inhalation], and then move back safely into myself [on exhalation]. [Having the exhalation not involve nourishment] lets me take a little break. It was a beautiful set up for [people with resistance to feeling nourishment like me] (+I^1,2,3^)”.

The six participants described widely varied experiences of the ocean tides meditation (Table 5). Instructions that worked well for some participants tripped others up. Lena and Alice wrestled with how to visualize tide coming in and going out with every breath. Following the instructions, Amruta and Kat breathed in nourishment and exhaled cleansing throughout the experience. For Kat inhalation and exhalation were linked to the ocean tides. Amruta focused only on her breath, ignoring the tide metaphor. The meditation object (specifically, the idea of the incoming tide bringing in nourishment) triggered resistance for Alice and Jade.

### 3.3. Essence-of-the-Ocean Meditation

#### 3.3.1. Meditation Overview

Table 6 summarizes key steps in the essence-of-the-ocean meditation structure, starting with preparation, followed by the main meditation, and a closing step to complete and prepare to transition out of the meditation.

#### 3.3.2. Why and for Whom Might this Meditation Be Beneficial?

This was the eighth and final meditation in the two-month series of weekly meditations that used some aspect of oceans as their meditation object (two of the other meditations were described in the preceding pages). This last one was the most open-ended and least complex meditation of the series. There were only two steps of preparation: a Sanskrit chant honoring the ocean (referred to as a mantra in the participant comments) and some simple forward bends. The vast majority of time (steps 3 through 7) involved being seated with eyes closed, calling to mind an ocean, drawing the essence of the ocean inside, and staying with the feeling, first with some movement and then again with only breath.

Who might this meditation be good for? The short preparation phase leaves more time in a half hour practice for the main meditation. Short preparation is fine for people who do not need much preparation for their mind to be ready to maintain attention on a meditation object. But short preparation can be insufficient for people whose attention benefits from longer preparation. Simple steps (in this meditation, chanting and then standing forward bends) can be ideal for some people, but other people are more able to keep their attention focused during meditation when they are doing complex practices that demand concentration, practices that combine many different activities at once. One size does not fit all.

No details about the ocean are specified (such as vastness or calm, waves or tide). Just call to mind an ocean. The teacher did not say “imagine you’re seated in front of the ocean”. He did not say “call to mind a particular positive experience of the ocean”. He did not say “call to mind that you’re on the ocean”. It just was “call to mind an ocean”. What comes to mind will be partially determined by each participant’s prior experiences with oceans, what is going on in their lives, and the state of their system that morning. The individual’s prior experiences with the recent vastness of the ocean meditations will also color their experience with this week’s meditation.

The chant was introduced three weeks ago at the very end of the meditation, where it was used to bring out of the vastness of the ocean. Then two weeks ago, that same chant was used in several of the steps. The sense of vastness will color the feeling of this chant regardless of whether people are aware of it, because their recent experience of that chant were associated with vastness.

Why might this meditation be beneficial? No specific quality of the ocean is prompted. There can be positive qualities associated with oceans such as beautiful, calm, vast, or deep. There can be negative qualities associated with oceans such as cold, destructive or dangerous. Because the meditation is so open, the potential benefits will depend upon what comes up for the person when they do the meditation.

#### 3.3.3. Participant Experiences

Melissa recently had a life-threatening experience in the ocean, which could have resulted in this being a frightening meditation experience. Fortunately, she discovered that she can do ocean meditations without those memories coming up. Melissa “had a near-drowning experience in Hawaii just a couple of years ago and it was a really scary one, where I really thought, ‘I’m going to be one of those stories (–O^BJECT^).’ It affects me if I go underwater snorkeling and things like that. I think about it then. But otherwise, it just really has not affected me with my meditations. Thank goodness because [oceans are] my favorite [meditations] (+O^BJECT^)”.

Analysis of the earlier meditation discussions in this article illustrated times when confusion with the instructions interfered with the person’s ability to keep their attention focused on the meditation. Ironically, even when the instructions work exceptionally well, the mind can get distracted. For Melissa, “it was really super easy to just go right into it. And then I sat there, as I got distracted thinking, ‘How was it so easy to just fall into that?’ And we’ve just barely gotten started. So I got distracted [over] why was it so easy, which is a little bit strange (–I^1,2,3^)”. The distraction was temporary. Melissa was able to return her attention to the ocean and get back on track. “I have not been doing the meditations this month or the last month. And so this was the first one and I absolutely just loved it. It’s such a nice meditation”.

What is going on in a person’s life can influence their meditation experience. Jade was on vacation by a beach the week of this meditation (+L^IFE^). “So it’s been interesting to do these meditations while here because we’re trying to decide if the math really works and we’re going to afford to keep [this vacation space]. So I’ve been able to work with the meditations and my relationship to the natural space here which is very different from [the city] where we live. The jury’s still out about what will happen next. But I felt like I was able to bring the peace of the ocean, the immensity of the ocean into me much more this week, starting with the chanting. This week, just doing the chanting and sitting quietly and making sure I had an ocean in view, I really had a very deep, very beautiful, peaceful experience and I think I really internalized it”.

For Jade the openness of the word ‘essence’ worked really well (+I^1,2,3^). “I like that word very much. I don’t know what other word you were thinking of using but just the essence of the ocean really allowed me a lot of latitude, I guess, a lot of room to smell it or taste it or whatever. It wasn’t directed”.

While Jade was smelling or tasting the beautiful peaceful ocean along her vacation property, Kat was being the entire Pacific Ocean. In an experience that was probably seeded by the earlier vastness of the ocean meditations (+O^BJECT^), Kat remarked the she still felt “a little jarred to be back in human form after being an ocean. I realized that a beach is such a tiny part of the ocean and so instead I was the entire Pacific Ocean. And I was interacting with the Earth and the sky and the moon, then down to individual particle level, and hosting life. I was being pure liquid and movement and temperature variations and being vast. It was extremely delicious. I loved how much time you gave us to just be in this state (+I^1,2,3^)”.

Lena enjoyed the brief preparation followed by long meditation. For Lena, “the small amount of movement and mantra at the beginning and then just going right into meditation really worked (+I^1,2,3^). It was easy for me. And I liked that because my [personal] practices usually have [a lot of] preparation (+L^IFE^). I found that I was able to instantly be in the ocean. I didn’t want to look at the ocean. I just wanted to be on the ocean or in the ocean”.

Lena’s mind was busy observing the experience she was having. “The qualities of [the ocean] were quite clear to me… There was language, so I felt like I had this witness saying, ‘Oh, the ocean is blue-green and it’s clear and I feel this motion.’ And so there was the observing of what I was experiencing and then there were the moments when I just felt like I was with the ocean, in the ocean, part of the ocean. It was really nice and it related to a piece of my personal practice which is about dissolving my own unique identity and not being separate”.

While she was doing the meditation, Lena thought, “oh, maybe these moments where I have language, I’m naming what I’m experiencing, is a different level of depth. And then when I’m actually being and feeling and experiencing myself as ocean, this is a different level of release”. She felt at some points “like I had the choice to let go of the thinking and just relax and just feel. So it was really interesting. The qualities of the ocean to being watery and fluid and clear and light were a really nice contrast because it’s dry and windy here. And I felt like, ‘Wow, I need to stay in this and not be affected by all of that.’ My ability to connect to ocean, or to dissolve me and be a part of the ocean, it was pretty direct. [It was] pretty clear to me that it was related [to stuff that’s going on for me personally] (+L^IFE^)”.

For Alice, complex instructions in a mediation help keep her mind focused. She is able to follow a complicated practice. Doing those things helps Alice’s mind have an experience of being directable, of being in a state of yoga. However, this essence-of-the-ocean meditation was very simple, and Alice’s mind ended up floating from idea to idea. Her mind wandered (–I^1,2,3^). She felt at the end of this meditation, “my mind just kept floating away into all sorts of things. I thought, wow, I think I’m missing the more intense [breathing techniques], the more directed and complicated [steps] and the longer [breath]. And then I would come back with the mantra which was useful”.

The meditation was a positive experience for Alice, but it was not of the same quality and depth that she can usually achieve in meditation. Having a lot going on in her life (including a newborn grandchild) also made it harder to keep attention focused (+L^IFE^). “I just stayed floating on the surface. It was just a little foggy. The ocean “did shift around a little bit. Mostly I just had a beautiful sense sort of like what’s behind a gem. It’s just a beautiful, soft, not necessarily the beach but turquoise blue. I was very aware of the temperature which was nice and warm and the water was clean and it was a nice level of saltiness. It felt very cleansing to bring it in and out. It was a very pleasant experience, but then other things would just come into my mind, maybe because so many things are going on in my life right now. It’s a little hard to not be distracted”.

Although simple practices are usually effective for her, Amruta “didn’t connect to today’s practice. It really fed more into my distracted energy. I don’t know if I came distracted or I just didn’t connect. Especially when we were doing the chanting with the arm movement and the breath, that really made me think, ‘Oh, this is just not working for me,’ because then I was trying to get it right – the inhalation, the exhalation, when does the chanting begin? How does the breath go? Because I was already not connected, it went into more frustration (–I^1,2,3^)”. She felt distracted until about step six and seven. “The ocean kept coming to me sporadically. It’s coming and going because I’m trying to invite it. And when I asked for it, it’s there. It’s clear, it’s blue, it’s vast, it’s deep. When you say essence, I’m able to bring it in. But it’s not natural today”.

Chanting in this meditation agitated Amruta and interfered with her ability to focus on the meditation (-I^1,2,3^). Amruta grew up in India “with a lot of Sanskrit chanting but it actually worked adversely for me because, for me, it was just a lot of repetition without connection. There are some mantras that I connect to easily because of the energy that comes with it or [the] meaning [that] sinks in for me or if [the words help] make me present in my body. But I’d say those instances are not many for me”. In today’s meditation, “the chanting actually was bringing me out of my body. I never began with a presence in my body. We began standing, then we went to sitting, then we went to chanting, then we were breathing and raising arms. It just kept scattering. For me, I connect to mantras only if I am able to feel the meaning and the essence of it in my body. Otherwise, they [are just] repetitive words for me that [distract me]”.

Although chanting had the effect of preventing Amruta from keeping her attention focused on the meditation object, chanting in this meditation was effective for Penelope. Starting the meditation with the mantra and a tiny bit of movement worked well for her. “I was able to just jump right in and have the experiences some other people have mentioned (+I^1,2,3^)”.

Meditation is an experience that unfolds over time. Penelope’s experience shifted about halfway into the meditation. “It’s like I almost had two different meditations today. The first meditation was really about the connecting to the vastness and already having planted that seed about vastness early on was l like, ‘oh, yeah,’ of course, that vast image was exactly what came up for me”.

A seemingly innocuous one-line instruction to ‘breathe in the essence and feel the essence inside of you’ totally changed Penelope’s experience. Steps 6 and 7 “brought me out and distracted me and turned up a bunch of chaos (–I^1,2,3^). This week, my state is a little [distracted] and so things got churned up (–S^os^)”. This was a temporary interruption to the meditation. Penelope was able to recover her attention. “And then the second half [of my meditation], was a very different image a very different feeling. It was just a bay, a very calm bay with all sorts of tropical fish. And it was just a completely different feeling and image of the ocean. It was just so beautiful it almost brought me to tears by the end”.

A core premise of Viniyoga is that yoga and meditation should be adapted to the individual. Yoga teachers who incorporate brief meditations as part of their group yoga classes are trained to carefully choose meditation objects and to structure the meditations in ways that are likely to be beneficial while trying to limit the likelihood of triggering a negative experience. Despite such precautions, there is always the possibility that someone doing a meditation will have an uncomfortable experience. Painful memories or associations may come up. For example, even though many people have strong positive feelings about oceans, Melissa’s near-drowning life experience could (but fortunately did not) result in her having a frightening essence-of-the-ocean meditation.

Doing a meditation can bring up issues or personal patterns the person meditating had not been aware of before the meditation. These kinds of realizations are not necessarily pleasant, but they can be very valuable. Grace is an experienced meditator and meditation teacher in the tradition of Viniyoga. When she did the essence-of-the-ocean meditation, something uncomfortable came up for her.

One of the previous ocean meditations (not included in this article) instructed participants to remember and feel the vastness of the ocean. Towards the end of that meditation, the instruction was to “explore the relationship between drop and ocean. You are a drop and you are sooo vast”. Grace resonated with the vast ocean meditation. “It fits in with the way I think of think of things like I tie it into the idea of spaces in the body and spaces in the mind which is something I’m always hammering in, in my own classes”. For Grace, “the drop is simply a synthesis of the vastness of the whole ocean. The whole ocean is in the drop. So there was no restriction. There was no feeling smaller or there was none of that because I see the drop as representing the ocean, just like my consciousness represents the universe’s consciousness (+O^BJECT^)”

Over the next week, Grace continued to reflect on the relationship between drop and ocean. During this week’s meditation, the vastness and the drop idea from a previous meditation showed up again in Grace’s current meditation experience (–O^BJECT^). This time, she found herself “stuck on the drop and I keep shifting from the drop to the vastness and I’m not resolving it. I’m watching the drop from someplace else and the drop has a color that does not mingle. There is no liquidity at all in that ocean. So I’m shifting from one place to the other and it’s very unsettling. It’s like a sense of loneliness with the drop and then the opposite with the vastness. So, it’s not very pleasant for some reason”.

Grace sensed the meditation was revealing something important about her life that she wanted to understand and deal with (–L^IFE^). At this point in the class discussion, the teacher observed “it sounds like there’s something that’s up for you [in your life]”. Grace agreed. In Viniyoga, even the experts who teach continue to study with a mentor. Bossart urged Grace to “meet with her mentor, to help her negotiate what’s coming up”.

The essence-of-the-ocean meditation had the most open-ended object of mediation of the three examples in this article. It had the shortest and least complex preparation and the longest time spent in the main meditation. All three of those characteristics contributed to the extreme diversity of experiences participants engendered (Table 7). It is almost as if they were doing completely different meditations. The simplicity contributed to Alice’s difficulty staying focused. The chanting distracted Amruta. Other participants loved the simplicity and the openness. The chanting worked well for Jade and Penelope. Things going on in Alice’s life influenced her meditation experience. Prior experiences with ocean meditations in the weekly class influenced Kat, Penelope, and Grace’s experiences. One size does not fit all.

## 4. Discussion

The goal in this article was to make the experience of doing meditation in the tradition of Viniyoga palpable. The cornucopia of approaches, systems, and definitions of meditation presents huge barriers to understanding and studying meditation as an intervention for health and well-being. This article was written for research scientists who study other forms of meditation interventions, for healthcare professionals who prescribe yoga or meditation as a form of complementary medicine for their patients, and for people who have tried mindfulness or other forms of meditation and are curious about how yoga-based meditation works.

The selection of participant quotations, explanations added by the authors, and ordering of presentation were approached from a strong analytical framework based on the authors’ study of Viniyoga, yoga anatomy, yoga philosophy, and years of personal experience teaching and taking the Therapeutic Application of Yoga classes. Many examples of the kinds of meditation objects were offered including how and why particular meditation objects might be chosen. The article referenced the five-dimensional yoga anatomy model of the human system to explain (1) why each person has a unique meditation experience, (2) why meditation can be considered a form of ethnomedicine, and (3) why Viniyoga meditations are designed for the individual.

The Therapeutic Application of Meditation class transcripts provided fertile ground for showing how each individual meditator engenders a unique personal experience. The ocean meditations were not iconic, classic, model meditations. They were designed to give meditation teachers and yoga therapists personal experiences with diverse meditation techniques, structures, and meditation objects. The series of discrete steps, many including visualization, the half-hour duration of the meditations as well as the capacity for self-observation the participants had developed over years of practicing yoga and meditation contributed to the richness of the descriptions that emerged.

The experiences participants described reveal meditation as an ongoing process of focusing attention on the meditation. The connection is not continuous. Attention fluctuates. For many participants, challenges and events happening in their lives showed up in the meditation experience. Some talked about how the state of their system at the time of the meditation interfered with their ability to focus. The structure or steps in each meditation were supportive of the experience for some participants yet that same structure impeded the experience for other participants. Affinity for, aversion to, and prior experiences with the meditation object influenced the meditation experience.

This article shows that even an identical meditation class yields unique experiences. Self-report of subjective experiences reveals how three identical meditation practices delivered live online resulted in unique subjective experiences for every participant. These practices and self-reports illustrate Viniyoga meditation experiences with different meditation objects.

Subjective experiences of meditating on something somewhat tangible such as qualities of the ocean are amenable to being recalled and described in rich detail. The uniqueness of individual experience with meditation illustrated throughout this article does not negate objective research on the mechanisms and effects of meditation, but it does clearly demonstrate that what happens for one person doing a meditation “intervention” is a unique result of the person interacting with the meditation instructions at whatever time of day and point in their lives they do the meditation.

Meditation is an umbrella term describing myriad approaches and practices. Theories and research on “mechanisms of effects of meditation” propose and test explanatory causal models. Logically extending the evidence of unique subjective meditation experiences presented in this article, it is likely that every meditation experience, not just Viniyoga meditations with a selected meditation objects, is unique. How should idea that every meditator has a unique experience impact the conduct and interpretation of scientific research on the effects of meditation?

Many other studies of subjective experiences of meditation are possible. The four factors of the individual this article focused on that influence how a meditation experience apply universally, but they are not exhaustive. Different meditation approaches, contexts, and audiences could also be studied. For example, one could study the experiences of students who attend weekly yoga classes that incorporate meditation. One could compare the experience of novice and expert meditators when they do an exemplar meditation. One could conduct case studies of yoga therapy clients discussing their experiences of meditation in their personal daily practice.

## 5. Conclusions

The authors analyzed self-reported experiences with three ocean meditations by nine long time meditation practitioners enrolled in a Therapeutic Applications of Meditation class. The goal was to illustrate some of the ways an individual interacting with the steps and instructions in a meditation engenders unique personalized meditation experience and outcomes. The presentation of self-reported subjective experiences showed how aspects of the individual influence what happened for them in a meditation.

Meditation is a unique subjective experience. Yoga therapists design a personal practice and meditation for a client based on the client’s needs, goals, and their Pañcamaya (their mind, body, breath, personality, and emotions). Yoga teachers who choose to do so design meditation for their students. Through the process of doing a meditation, each person’s human system (their mind, body, breath, personality, and emotions) interacts with the meditation steps and instructions, engendering a unique personal experience. What will emerge is not precisely known before doing the practice. An experience unfolds. Observing what happens can yield self-knowledge and insights.

It is not necessary to understand why meditation works for it to have an effect, just like one does not need to know why being in nature feels good in order to receive the health benefits of being in nature. Ecotherapy research shows that spending time in nature, and even looking at pictures of nature brings health benefits. People who do so are more likely to experience pleasant feelings of calm and harmony, and a sense of increased energy and vitality [17]. Physiologically there may be reduced blood pressure, reduced cortisol levels, reduced pain sensitivity, and more relaxed breathing. In the same vein, meditating on different meditation objects activates some of the qualities of that object in us.

Simply reading explanations of what yoga-based meditation is does not convey understanding of what it is like to meditate. Reading scientific research about the mechanisms and effects of yoga-based meditation does not convey an understanding of what it feels like to meditate. Reading the instructions for a meditation does not convey an understanding of what it would be like to experience the meditation. Through the presentation of subjective perspectives from long-time practitioners of yoga-based meditation, this article provides a way to communicate to the reader a tangible sense of what it is like to experience Viniyoga meditations.

## Figures and Tables

**Table 1:** Ocean meditations and participants.

	Deep Calm	Tides	Essence	Total Sessions
Alice	X	X	X	3
Amruta	X	X	X	3
Lena	X	X	X	3
Kat	X	X	X	3
Jade	X	X	X	3
Penelope		X	X	2
Iris	X			1
Grace			X	1
Melissa			X	1
n	6	7	7	

**Table 2:** Calm-ocean-depths meditation structure.

PREPARATION
Set an intention “I am calm”. Seated: Gradually introduce an intricate sequence of breath control, finger movements, arm movements, and forward bends, one step at a time. After each breath in the sequence, pause after exhalation and mentally repeat “I am calm”.
MAIN MEDITATION
Have the sense of deep ocean: very still. Allow attention to rise to the surface where a giant storm is coming. Cycle through two or three rounds of still deep ocean to stormy surface to have a sense of the contrast.
RE-VISIT
Which part of you is the deep ocean and which part of you is the surface? Answer with an experience.

**Table 3:** Calm-ocean-depths summary.

**ALICE**	**IRIS**	**JADE**
It became very personal and was safe easy to feel where the storm calm are in my body.	It was reflective of my life and experiences. I was in, but detached from the storm.	My mind came up with a place (Gaudi Park) that was like deep ocean and storm.
**LENA**	**AMRUTA**	**KAT**
Each time I came up to the surface, the storm was less threatening.	There was turbulence but no storm. I am less affected by turbulence in my life now.	I became the storm, loving power and intensity. Deep calm was OK but not as fun.

**Table 4:** Ocean tides meditation structure.

PREPARATION
Call to mind: The ocean tide rises. Water comes in. So nourishing. Ocean tide goes out, so cleansing.
MAIN MEDITATION
Call to mind: The ocean tide rises. Water comes in. So nourishing. Ocean tide goes out, so cleansing.
Seated again: Inhale, the ocean tide comes in. Pause after inhalation. Feel high tide. Exhale and the ocean tide goes out. Pause after exhalation. Feel low tide. (An option is offered: if you prefer, the tide can come in and go out over several breaths rather than in each breath.)
TRANSITION
Chant (om sāgarāya namaḥ).

**Table 5:** Ocean tides summary.

ALICE	AMRUTA	JADE
Tides changing each breath confused me. It started out great until I wondered what was nourishing about a wave.	My mind was crowded so I just linked nourishing and cleansing with my breath instead of visualizing the tide.	The ocean water was not clean, so I skipped feeling nourished by the incoming tide and focused on the cleansing part.
LENA	PENELOPE	KAT
I appreciated the cleansing and nourishing. But the instructions distracted me.	At the beginning, little fish visited me. Then they went out with the next wave.	I would tentatively inhale nourishment, then enjoy the respite of exhaling cleansing.

**Table 6:** Essence-of-the-ocean meditation structure.

PREPARATION
Chant (om sāgarāya namaḥ). Standing: forward bends, pausing after inhalation and exhalation.
MAIN MEDITATION
Seated: call to mind an ocean. On inhalation, reach for the ocean. On exhalation, draw in the essence of the ocean while chanting. Stay with the feeling.
TRANSITION
Chant (om sāgarāya namaḥ).

**Table 7:** Essence-of-the-ocean summary.

ALICE	MORGAN	JADE
It was very pleasant, but hard not to be distracted.	I absolutely just loved it.	The chanting worked. I had a peaceful, beautiful experience.
LENA	PENELOPE	KAT
The qualities of the ocean were clear. I shifted between observing and experiencing.	At first, it was about vastness Later it was beautiful and calm	I was the entire Pacific Ocean, interacting with the earth and moon and life.
AMRUTA	GRACE	
Trying to chant fed into my distracted energy. I was sporadically with the ocean.	Shifting between the vast ocean and a single drop brought up loneliness for me this week.	

## Supplementary Materials

The following are available online at: http://carrieheeter.com/publications/ocean/s1.pdf, Therapeutic Application of Meditation Class Discussion Raw Transcripts and http://carrieheeter.com/publications/ocean/s2.pdf, Ocean Meditation Practice Details.
